# Supplementary material for: In Vivo Verification of Treatment Source Dwell Times in Brachytherapy of Postoperative Endometrial Carcinoma: A Feasibility Study
Source: J Pers Med. 2022 May 31;12(6):911. doi: 10.3390/jpm12060911 (PMC9224704; doi:10.3390/jpm12060911)
Supplement: Supplementary file 1 [file jpm-12-00911-s001.zip › jpm-1682172-supplementary.pdf]

Supplementary material

Linearity of PSD total counts with absorbed dose

Figure S1 shows the graphical data of the results obtained.

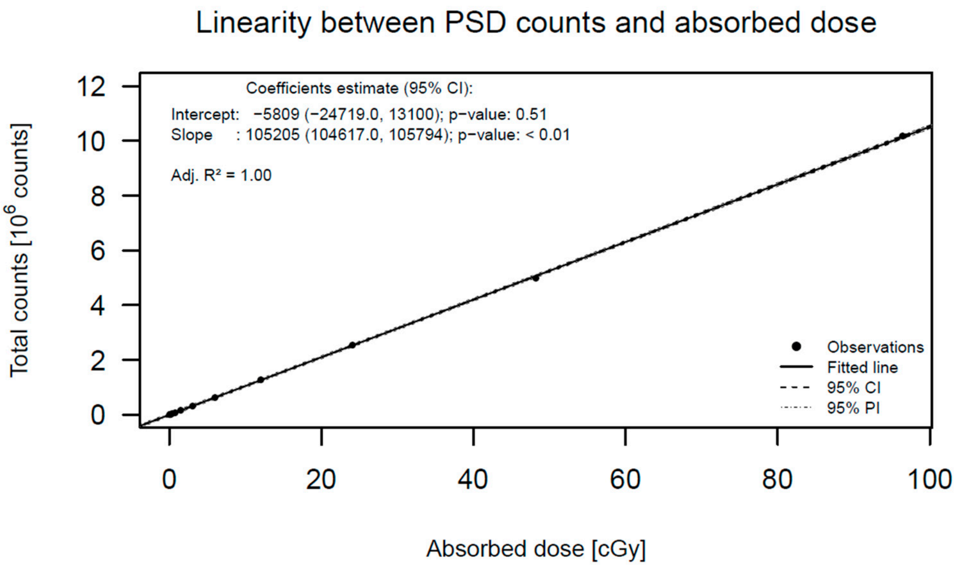

Figure S1. Linear regression of total counts with absorbed dose.

Repeatability

Tables S1-6 report the results obtained for the different combinations of irradiation time and acquisition frequency.

Table S1. Data analysed of 10 irradiations of 10 s corresponding to the repeatability test. (10Hz)[1]

PSD[2] count rate [average counts/s]	Repeatability[3] [%]	PSD[2] signal [total counts]	Repeatability[3] [%]	PSD[2] measured dwell time [s]	Repeatability[3] [%]
41535	0.4	415480	1.9	10.0	0.2
41328	0.1	409239	0.4	10.0	0.2
41370	0.0	401289	1.6	9.8	1.8
40894	1.2	405076	0.6	10.1	1.2
41261	0.3	404378	0.8	10.0	0.2
41523	0.3	402841	1.2	9.9	0.8
41357	0.1	405203	0.6	10.0	0.2
41640	0.6	416552	2.2	10.0	0.2
41354	0.1	413537	1.4	10.0	0.2
41521	0.3	402934	1.2	10.0	0.2

[1] Corresponds to a detection acquisition frequency of 10 Hz.
[2] PSD: Plastic Scintillator Dosimeter
[3] Repeatability = 100 * $|x_i - x_{mean}|/x_{mean}$

Table S2. Data analysed of 10 irradiations of 30 s corresponding to the repeatability test. (10Hz)[1]

PSD[2] count rate [average counts/s]	Repeatability[3] [%]	PSD[2] signal [total counts]	Repeatability[3] [%]	PSD[2] measured dwell time [s]	Repeatability[3] [%]
41423	0.1	1238565	0.2	29.9	0.3
41229	0.4	1232865	0.3	30.0	0.1
41321	0.2	1239605	0.2	30.0	0.1
41382	0.0	1237358	0.1	30.0	0.1
41594	0.5	1239773	0.3	30.0	0.1
41422	0.1	1238602	0.2	30.0	0.1
41438	0.1	1239092	0.2	30.0	0.1
41384	0.0	1233293	0.3	30.0	0.1
41401	0.0	1237950	0.1	30.0	0.1
41378	0.0	1228976	0.6	29.9	0.3

[1] Corresponds to a detection acquisition frequency of 10 Hz.

[2] PSD: Plastic Scintillator Dosimeter

[3] Repeatability = 100 * $|x_i - x_{mean}|/x_{mean}$

Table S3. Data analysed of 10 irradiations of 300s corresponding to the repeatability test. (10Hz)[1]

PSD[2] count rate [average counts/s]	Repeatability[3] [%]	PSD[2] signal [total counts]	Repeatability[3] [%]	PSD[2] measured dwell time [s]	Repeatability[3] [%]
41613	0.1	11901498	1.3	300.0	0.0
41795	0.3	12212661	1.3	300.0	0.0
41811	0.4	12275719	1.8	299.9	0.0
41772	0.3	12013502	0.3	299.9	0.0
41678	0.0	12111921	0.5	299.9	0.0
41629	0.1	12039130	0.1	299.9	0.0
41615	0.1	12072678	0.1	300.0	0.0
41590	0.2	12057017	0.0	300.0	0.0
41469	0.5	11814692	2.0	300.0	0.0

[1] Corresponds to a detection acquisition frequency of 10 Hz.

[2] PSD: Plastic Scintillator Dosimeter

[3] Repeatability = 100 * $|x_i - x_{mean}|/x_{mean}$

Table S4. Data analysed of 10 irradiations of 10 s corresponding to the repeatability test. (20Hz)[1]

PSD[2] count rate [average counts/s]	Repeatability[3] [%]	PSD[2] signal [total counts]	Repeatability[3] [%]	PSD[2] measured dwell time [s]	Repeatability[3] [%]
21224	0.6	327868	6.7	9.95	0.5
21363	0.0	312028	1.6	10.05	0.5
21403	0.2	287054	6.5	10.00	0.0
21428	0.3	274302	10.7	10.05	0.5
21416	0.3	311241	1.3	9.95	0.5
21307	0.3	331597	8.0	10.05	0.5
21430	0.3	302826	1.4	10.05	0.5
21392	0.1	302658	1.5	10.05	0.5
21367	0.0	306687	0.2	9.95	0.5
21287	0.3	315398	2.7	9.90	1.0

[1] Corresponds to a detection acquisition frequency of 20 Hz.
[2] PSD: Plastic Scintillator Dosimeter
[3] Repeatability = 100 * | x_i - x_{mean} | / x_{mean}

Table S5. Data analysed of 10 irradiations of 3 s corresponding to the repeatability test. (20Hz)[1]

PSD[2] count rate [average counts/s]	Repeatability[3] [%]	PSD[2] signal [total counts]	Repeatability[3] [%]	PSD[2] measured dwell time [s]	Repeatability[3] [%]
20640	0.2	117691	2.4	2.95	3.8
20500	0.8	121059	0.4	3.05	0.5
20670	0.0	124048	2.9	3.10	1.1
20850	0.9	123106	2.1	3.10	1.1
20670	0.0	121996	1.2	3.10	1.1
20750	0.4	118425	1.8	3.10	1.1
20670	0.0	124060	2.9	3.00	2.1
20690	0.1	115903	3.9	3.05	0.5
20620	0.3	117608	2.5	3.05	0.5
20670	0.0	122124	1.3	3.15	2.8

[1] Corresponds to a detection acquisition frequency of 20 Hz.
[2] PSD: Plastic Scintillator Dosimeter
[3] Repeatability = 100 * | x_i - x_{mean} | / x_{mean}

Table S6. Data analysed of 10 irradiations of 1 s corresponding to the repeatability test. (20Hz)[1]

PSD[2] count rate [average counts/s]	Repeatability[3] [%]	PSD[2] signal [total counts]	Repeatability[3] [%]	PSD[2] measured dwell time [s]	Repeatability[3] [%]
20643	0.2	45415	13.6	1.10	5.3
20518	0.4	39017	2.4	1.05	0.5
21046	2.2	37882	5.2	1.00	4.3
20405	0.9	42909	7.3	1.05	0.5
20471	0.6	34820	12.9	1.00	4.3
20827	1.1	35343	11.6	1.00	4.3
20561	0.2	37070	7.3	1.00	4.3
20472	0.6	43058	7.7	1.10	5.3
20674	0.4	43483	8.8	1.05	0.5
20335	1.3	40751	1.9	1.10	5.3

[1] Corresponds to a detection acquisition frequency of 20 Hz.
[2] PSD: Plastic Scintillator Dosimeter
[3] Repeatability = 100 * | x_i - x_{mean} | / x_{mean}

Long-term Stability

Table S7 summarises the results obtained for the long-term stability of the PSD count rate. The relative standard deviation of the PSD sensitivity with count rate varied less than 1% demonstrating the high stability of the PRO-DOSE with cumulative absorbed dose and its low absorbed dose rate dependence.

Table S7. Data of the PSD count rate corresponding to the long-term stability test.

**RAKR**[1] [$mGy \cdot m^2 \cdot h^{-1}$]	**PSD**[2] **average count rate** [counts/s]	**Absorbed dose rate at PSD**[2] [cGy/s]	**Sensitivity**[3] [10^3·(counts/s)/(cGy/s)]
41.19	59640	0.700	85.2
38.52	55840	0.654	85.4
36.06	51770	0.612	84.5
33.79	49230	0.574	85.8
31.60	46270	0.537	86.2
27.73	39620	0.471	84.1
25.70	37070	0.436	84.9
23.65	33640	0.402	83.8

[1] RAKR: Reference Air Kerma Rate.
[2] PSD: Plastic Scintillator Dosimeter
[3] Sensitivity = PSD count rate /absorbed dose rate.

Table S8 shows the long-term stability of the PSD total counts. The relative standard deviation of the PSD sensitivity with total counts was lower than 1.2% demonstrating the good linearity with absorbed dose of the dosimeter.

Table S8. Data of the PSD total counts corresponding to the long-term stability test.

**RAKR**[1] [$mGy \cdot m^2 \cdot h^{-1}$]	**PSD**[2] **average total counts** [counts]	**Absorbed dose at PSD**[2] [cGy]	**Sensitivity**[3] [10^3·(counts)/(cGy)]
41.19	1205456	14.0	86.2
38.52	1130818	13.1	86.4
36.06	1036961	12.2	84.7
33.79	992498	11.5	86.5
31.60	941254	10.7	87.7
27.73	805213	9.4	85.5
25.70	748782	8.7	85.8
23.65	679296	8.0	84.6

[1] RAKR: Reference Air Kerma Rate.
[2] PSD: Plastic Scintillator Dosimeter
[3] Sensitivity = PSD count rate /absorbed dose rate.

Signal-to-noise ratio (SNR)

Table S9 summarises the signal-to-noise ratio results obtained during the period of long-term stability analysis.

Table S9. Data of the PSD and background count rate corresponding to the signal-to-noise test.

**RAKR**[1] $[mGy \cdot m^{2} \cdot h^{-1}]$	**PSD**[2] **signal amplitude [average counts/s]**	**Noise [average counts/s]**	**SNR**[3]
41.19	59630	1170	51.0
38.52	55840	1120	49.9
36.06	51760	1130	45.8
33.78	49220	1160	42.4
31.60	46260	1140	40.6
27.73	39620	1160	34.2
25.70	37070	1140	32.5
23.65	33640	1150	29.3

[1] RAKR: Reference Air Kerma Rate.
[2] PSD: Plastic Scintillator Dosimeter
[3] Signal-to-Noise Ratio: SNR = (amplitud)/(noise)

Linearity of PSD total counts with absorbed dose

Table S10 summarises the results obtained in the linearity test for the PSD total counts with absorbed dose.

Table S10. Data of the irradiations corresponding to PSD total counts linearity test.

**Nominal dwell time** [s]	**PRO-DOSE dwell time measured** [s][1]	**Sensitivity**[2] $[10^{3}$ counts/cGy]	**Deviation of total counts from predicted in regression** [%]
204.8	204.8	105.5	-0.3
102.4	102.4	103.6	1.4
51.2	51.2	104.9	0.1
25.6	25.6	104.9	-0.2
12.8[3]	12.8	103.8	0.5
6.4	6.5	105.4	-2.0
3.2	3.2	105.5	-4.0
1.6	1.6	104.2	-6.4
0.8	0.8	102.9	-12.7
0.4	0.4	103.8	-28.4
0.2	0.2	100.6	-56.7
0.1	0.1[3]	90.8	-120.0

[1] Average of three consecutive measurements performed with an acquisition frequency of 10 Hz.
[2] Sensitivity = PSD counts /absorbed dose.
[3] Times below this value are not used in vaginal cylinder brachytherapy (VCBT).

Linearity of PSD count rate with absorbed dose rate

Table S11 summarises the results obtained in the linearity test for the PSD count rate as a function of absorbed dose rate.

Table S11. Data of the irradiations corresponding to the PSD count rate linearity test

**Absorbed dose rate at PSD**[1] [cGy/s]	**PRO-DOSE count rate**[2] [counts/s]	**Sensitivity**[3] [10^3 counts/s)/(cGy/s)]	**Deviation of count rate from predicted in regression** [%]
0.700	59630	85.2	-0.1
0.654	55840	85.4	-0.3
0.612	51760	84.5	0.7
0.574	49220	85.8	-0.8
0.537	46260	86.2	-1.2
0.471	39620	84.1	1.2
0.436	37070	84.9	0.2
0.402	33640	83.8	1.6

[1] PSD: Plastic Scintillator Dosimeter

[2] Average of three consecutive measurements performed with an acquisition frequency of 10 Hz.

[3] Sensitivity = PSD count rate /absorbed dose rate.

Precision of dwell time measurements in patient treatments

The behaviour of the PRO-DOSE system in the whole range of treatment dwell times is depicted in Figure S2 and shows good linear agreement between planned and measured dwell times.

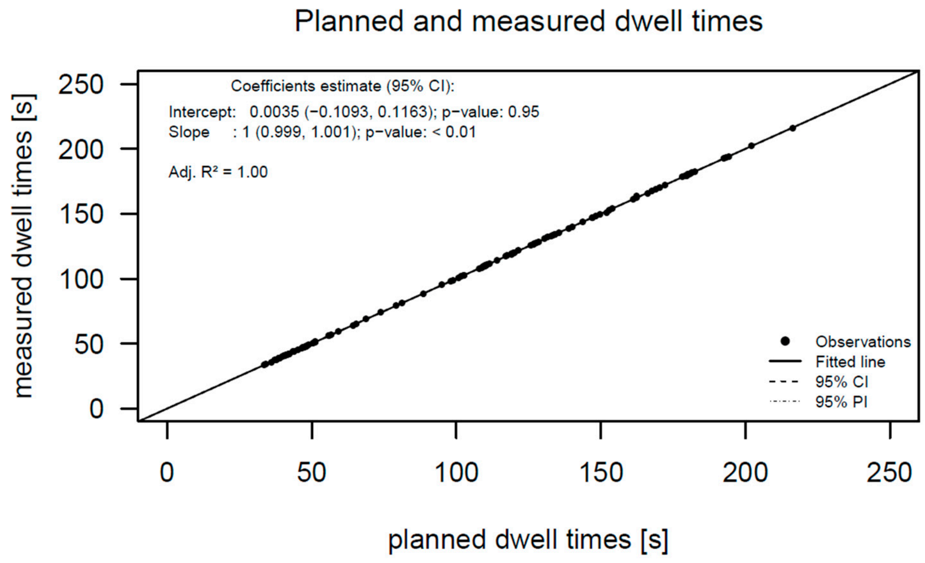

Figure S2. Linearity of planned and measured dwell times in vaginal cylinder brachytherapy (VCBT).

Analysing the data as a quotient between measured and planned dwell times gives us a clearer information of the deviation as shown in Figure S3.

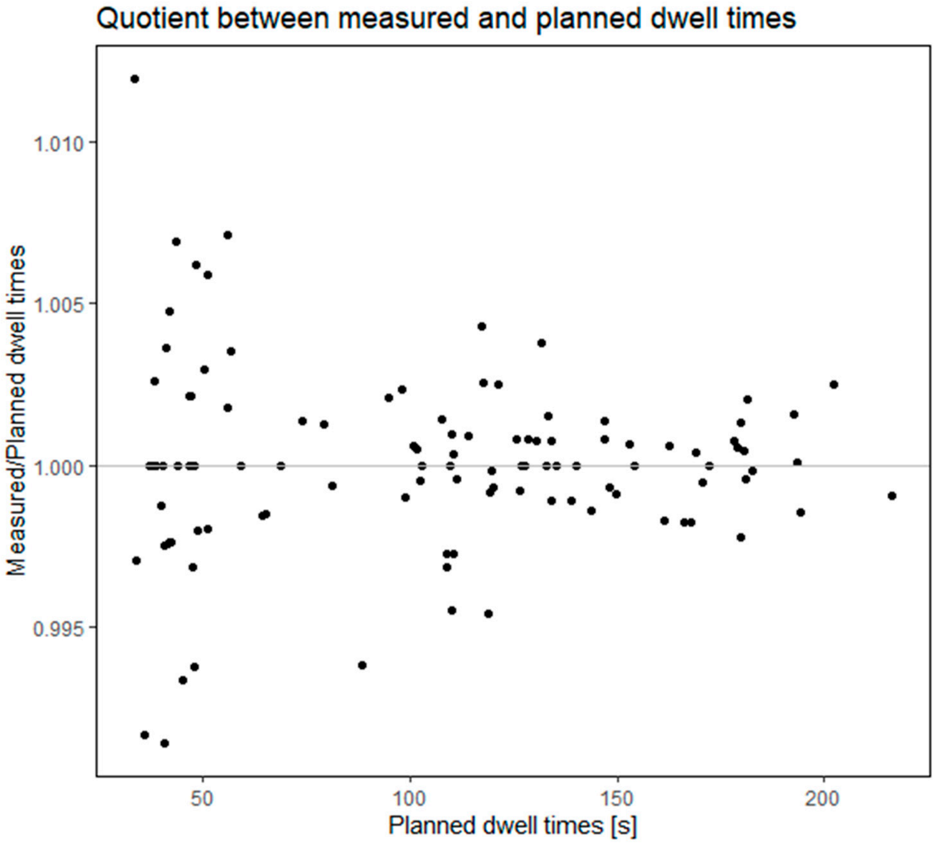

Figure S3. Quotient between measured and planned dwell times.
